# Mapping PRNP Polymorphisms in Portuguese Serra da Estrela Ovine Populations: Insights into Scrapie Susceptibility and Farm Animal Improvement

**DOI:** 10.3390/ani15182750

**Published:** 2025-09-20

**Authors:** Soraia Rodrigues, Guilherme Moreira, Sérgio Santos-Silva, Sara Gomes-Gonçalves, Maria Aires Pereira, Alexandra Baptista, Rita Cruz, Fernando Esteves, João R. Mesquita

**Affiliations:** 1 School of Medicine and Biomedical Sciences (ICBAS), Universidade do Porto (UP), 4050-313 Porto, Portugal; up201707495@up.pt (S.R.); gmoreiravet@gmail.com (G.M.); up202110051@edu.icbas.up.pt (S.S.-S.); smggoncalves@icbas.up.pt (S.G.-G.); 2 Escola Superior Agrária de Viseu, Instituto Politécnico de Viseu, 3500-631 Viseu, Portugal; mapereira@esav.ipv.pt (M.A.P.); alexabaptista@esav.ipv.pt (A.B.); rcruz@esav.ipv.pt (R.C.); festeves@esav.ipv.pt (F.E.); 3 CERNAS-IPV Research Centre, Instituto Politécnico de Viseu, Campus Politécnico, Repeses, 3504-510 Viseu, Portugal; 4 Global Health and Tropical Medicine, GHTM, Associate Laboratory in Translation and Innovation Towards Global Health, LA-REAL, Instituto de Higiene e Medicina Tropical, IHMT, Universidade NOVA de Lisboa, UNL, Rua da Junqueira 100, 1349-008 Lisboa, Portugal; 5 Universidade de Trás-os-Montes e Alto Douro, Quinta de Prados, 5000-801 Vila Real, Portugal; 6 Epidemiology Research Unit (EPIUnit), Instituto de Saúde Pública da Universidade do Porto, Rua das Taipas 135, 4050-600 Porto, Portugal; 7 Laboratory for Integrative and Translational Research in Population Health (ITR), Rua das Taipas 135, 4050-600 Porto, Portugal; 8 Associate Laboratory for Animal and Veterinary Science (AL4AnimalS), 1300-477 Lisboa, Portugal; 9 Centro de Estudos de Ciência Animal (CECA), Instituto de Ciências, Tecnologias e Agroambiente (ICETA), Universidade do Porto (UP), 4051-401 Porto, Portugal

**Keywords:** *PRNP* gene, scrapie resistance, sheep, Serra da Estrela, single nucleotide polymorphisms (SNPs), animal improvement

## Abstract

Scrapie (classical and atypical) susceptibility in sheep is strongly influenced by *PRNP* gene polymorphisms. In Portugal, limited data exist for native breeds such as Serra da Estrela, despite their relevance to animal conservation and food production. The full coding region of *PRNP* gene of 92 Serra da Estrela sheep was sequenced and SNP frequencies were analysed. The predicted functional impact of nonsynonymous SNPs was assessed using PolyPhen-2 and AMYCO. A total of 27 SNPs were identified, including 20 nonsynonymous variants. Thirteen major haplotypes were observed. The ARR allele, which provides resistance to classical scrapie, was present in 58.7% of the population, with 18.5% of animals being homozygous. Several previously unreported SNPs were identified, and their impact on prion protein aggregation propensity and structure was explored. The high frequency of the ARR allele without full ARR fixation suggests that no selective breeding for scrapie resistance has been applied. These results support the adoption of gradual selection strategies that preserve genetic variability and promote farmer compliance, while increasing classical and atypical scrapie resistance.

## Simple Summary

Scrapie is a fatal disease in sheep that can be influenced by natural genetic variation. We analysed the genetic profile of Serra da Estrela sheep, a native Portuguese breed to understand how common protective or susceptible gene variants are in this population. We found that many animals carried a genetic form that provides resistance to classical scrapie, while the most susceptible variants were absent. Several new gene mutations were also detected, some of which may play a role in how the disease develops. These results suggest that gradual and balanced breeding strategies could improve resistance to scrapie in Serra da Estrela sheep without reducing their genetic diversity. Strengthening the health of this breed not only improves animal welfare but also helps preserve an important cultural and economic resource for Portugal.

## 1. Introduction

Classical scrapie is the oldest known transmissible spongiform encephalopathy (TSE), with historical records dating back to the 18th century in Europe. It affects sheep and goats and is characterised by progressive neurodegeneration, often leading to behavioural changes, pruritus, ataxia, and ultimately death. The name “scrapie” derives from the intense itching behaviour observed in affected animals, who frequently scrape their bodies against surfaces [1,2].

As a prion disease, scrapie is caused by the accumulation of an abnormal isoform of the prion protein (PrP^Sc^), which acts as the infective agent. In 1998 an unusual non-contagious form of scrapie, affecting older animals, was identified in Norway for the first time and was designated atypical scrapie (or Nor98). The prion protein is encoded by the *PRNP* gene and genetic susceptibility to scrapie in sheep has been strongly linked to polymorphisms within this gene, making its study essential for understanding disease dynamics and improving breeding strategies for resistance [1,2].

Current evidence shows that key single-nucleotide changes in the *PRNP* gene occur mainly at codons 136 (A > V), 154 (R > H), and 171 (R > Q/H) [3,4]. Alterations at codons 141 and 154 have also been linked to different presentations of classical and atypical scrapie, most likely because of their effect on prion protein conformation [5]. The substitution A → V at codon 136 has been associated with a higher risk of developing classical scrapie, while the Q→R change at codon 171 is considered protective in sheep [3,4]. It is believed that the ancestral *PRNP* gene was A_136_R_154_Q_171_/A_136_R_154_Q_171_ (hereafter ARQ/ARQ) in sheep [1]. The five most common haplotypes are ARR, ARQ, AHQ, ARH, and VRQ, and the genotypes that result from their different pairings are grouped into five resistance categories. More recently, additional amino acid substitutions in certain nucleotide positions have also been reported to confer resistance to classical scrapie, even in animals carrying the ARQ/ARQ background [1,2].

Numerous investigations have examined *PRNP* gene variation in sheep from different breeds and geographical regions, frequently assessing its connection with productive or phenotypic characteristics. In China, research has examined the testis-specific *PRNP* gene in Chinese and Mongolian sheep [6], as well as phenotypic traits in different Chinese breeds [7], Xinjiang local breeds [8], and Tan sheep from Ningxia [9]. Other studies have focused on specific traits, such as milk production in Latxa dairy sheep [10] and Spanish Churra [11], reproductive traits in German breeds [12], pathogenesis and neuropathological phenotypes [13], the Rasa Aragonesa breed from Spain [14], and general polymorphisms in Baltic breeds [15]. Additional country-specific studies have been conducted in Greece [16], Ireland [17], Brazil [18], the Iberian Peninsula [19], Italy [20], Türkiye [21], and the native sheep breed of Palestine [22]. Within China, further studies have examined sheep from Inner Mongolia [23] and Northwest China [24]. In Africa, only a few studies have reported *PRNP* gene polymorphisms, including Algeria [25], Ethiopia [26], Morocco [27], and four West African sheep populations: Burkina-Sahel, Djallonke, Mossi, and Touareg [28].

Previous studies in Portugal, in 2006 and in 2010, investigated the distribution of genetic polymorphisms in the PrP locus in Portuguese sheep; however, the methods used provided information only in the target codons 136, 154, and 171. These studies sampled sheep of various Portuguese breeds, with Serra da Estrela being the one with the higher frequency of ARR alleles while having very low frequencies of the undesirable VRQ [29,30]. Between 2002 and 2008, a total of 275,259 small ruminants were screened for atypical scrapie, and 326 sheep tested positive. The cases were associated with a variety of genotypes, most commonly ARR/AFRQ, followed by ARR/ARR, ARQ/AFRQ, and AFRQ/AFRQ [31]. From 2002 to 2021, atypical scrapie cases were reported in 24 countries, with Portugal recording the highest number (696), followed by France (571) and the United Kingdom (368) [32].

This study aimed to characterise the *PRNP* gene in the Serra da Estrela breed in Portugal not only to assess the distribution of genotypes but, also, to investigate other polymorphisms in the *PRNP* gene, specifically mutations like L141F, which is associated with atypical scrapie [33], and other potential susceptible or protective polymorphisms. To achieve this, we analysed the genotype and allele frequencies of PRNP polymorphisms in a sample of 92 Serra da Estrela sheep and compared them to the wild-type sequence. Additionally, the potential biological impact of the identified nonsynonymous single-nucleotide polymorphisms (SNPs) was also evaluated, with particular emphasis on prion protein structure and function.

## 2. Materials and Methods

### 2.1. Geographical Location and Study Population

This study was conducted in the Serra da Estrela region, the highest mountain range in mainland Portugal, reaching elevations of up to 2000 m above sea level. It is also the coldest region in the country, with average minimum temperatures ranging from −0.1 °C to 12 °C and maximum temperatures between 6 °C and 22 °C. From March to October, daytime temperatures typically exceed 10 °C [34]. The study focused on Serra da Estrela sheep registered in the breed’s genealogical book, which is overseen by ANCOSE (National Association of Serra da Estrela Sheep). This native breed, protected by a designation of origin, is exclusively raised in the Serra da Estrela region (approximately 40°26′8.84″ N, 51°59.94″ W). These animals are characterised by minimal mobility, remaining confined to farm premises during the day and sheltered at night, making them ideal for structured and geographically stable disease monitoring. The Last Census (2020) reported a total of 18,600 sheep of this breed [35]. Only sheep of the Serra da Estrela breed were included in this study.

### 2.2. Sample Collection

A total of 92 (*n* = 92) animals were included in this study. Brain tissue samples were collected from 23 female sheep originating from the centre region of Portugal. These samples were obtained post-mortem during routine slaughter for meat production in March 2024. The samples were transported that same day to the laboratory under refrigeration (4 °C) and subsequently stored at −20 °C until processed. Additionally, 72 blood samples, obtained as remnants from the Brucellosis control program in the same region of Portugal, were also used. Briefly, remnants from the 5 mL blood collected in EDTA tubes from the jugular vein of 72 sheep (all females) were randomly chosen in each of 12 different farms in the centre region of Portugal to reach six samples per farm. Since three blood samples were fully clotted before processing, these samples were excluded from the study, resulting in a total of 69 blood samples. The whole blood samples were stored at −20 °C prior to DNA extraction.

All procedures for animal handling and sample collection followed the authorisations granted by the Órgão Responsável pelo Bem-estar Animal da ESAV (ORBEA-ESAV), in accordance with the applicable ethical and legal guidelines (license number: 03.01/ORBEA/2024; approval date: 22 March 2024).

### 2.3. Nucleic Acid Extraction

Brain DNA extraction was performed using a modified QIAamp^®^ DNA Mini Kit (Qiagen, Valencia, CA, USA). A total of 420 μL of lysis buffer along with 25 μL of proteinase K solution was added to the corresponding Eppendorf tube. The samples were vortexed for 30 s to ensure proper mixing, centrifuged at 6000× *g* for 2 min, and incubated at 57 °C for 15 min to facilitate lysis. A 350 μL aliquot of the resulting supernatant was transferred to a fresh microcentrifuge tube and mixed with an equivalent volume of RTL buffer. The tubes were vortexed again for 30 s and pulse-centrifuged, and subsequent steps were carried out in accordance with the QIAamp^®^ DNA Mini Kit protocol using an automated QIAcube system (Qiagen^®^ GmbH, Hilden, Germany).

Blood DNA extraction was performed using the PurePrep96 magnetic-bead-based extraction system (MolGen^®^, Veenendaal, The Netherlands). Whole blood in EDTA tubes was resuspended and 200 μL of sample was used with the kit BioExtract^®^ SuperBall^®^ (MolGen^®^, Veenendaal, The Netherlands).

### 2.4. Polymerase Chain Reaction (PCR) and DNA Sequencing

To identify polymorphic sites in the *PRNP* gene, PCR was carried out using a single pair of primers that amplified the 771 bp open reading frame (ORF), corresponding to the same region of the *Ovis aries* PRNP sequence available in the NCBI database (Gene ID: EF153678): PRNP-Forward (5′-CATTTATGACCTAGAATGTTTATAGCTGATGCCA-3′) and PRNP-Reverse (5′-TTGAATGAATATTATGTGGCCTCCTTCCAGAC-3′) [3].

The 25 µL PCR mixture was obtained with the Takara Bio Inc. kit “TaKaRa LA Taq^®^ DNA Polymerase” kit (Takara Bio Inc., Otsu, Japan), and 2 μL of genomic DNA was used in each reaction, 0.6 µM of each primer, 2.5 mM dNTPs, and 1.25 units of TaKaRa La Taq^®^ DNA polymerase in the 10X LA PCR Buffer II with 2.5 mM added MgCl_2_. The PCR conditions were 96 °C for 5 min, 35 cycles of denaturation at 96 °C for 30 s, 57 °C for 15 s, 72 °C for 1 min 30 s, and final extension of 72 °C for 4 min. Electrophoresis using agarose gel with a 1 kb DNA ladder marker was conducted to confirm the gene was correctly extracted and amplified.

Amplicons of the expected size (771 bp) were purified using the Exo/SAP Go PCR purification kit (Grisp^®^, Porto, Portugal). After purification, bidirectional sequencing was performed using the Sanger sequencing method. Alignment and deconvolution of Sanger chromatogram trace files were performed using Tracy 0.7.6 (GEAR-Genomics) [36]. Sequence editing was conducted with the BioEdit Sequence Alignment Editor v7.1.9 (Ibis Biosciences), and alignment and comparison of SNPs and their translated proteins was conducted using MEGA-X version 10.2.6 software.

### 2.5. Statistical Analysis

Haplotype distributions of the *PRNP* gene were obtained using DNA SNP Version 6.12. Hardy–Weinberg exact test was conducted using R v4.2.2. Genotype differences, allele, and haplotype frequencies of the *PRNP* gene were analysed using Microsoft Office 365 Excel v2506.

### 2.6. Evaluation of Nonsynonymous SNPs in the PRNP Gene

Evaluation of 20 nonsynonymous SNPs in the *PRNP* gene was carried out using the predictive online software PolyPhen-2 v2.0 (http://genetics.bwh.harvard.edu/pph2/ accessed on 3 July 2025)), a tool for annotating nonsynonymous SNPs and predicting damaging effects of missense mutations in humans, which has been used before to assess SNPs in sheep [3,37]. The evaluation of the effect of mutations on the aggregation properties of ovine prion protein was explored using AMYCO [38]. AMYCO score relates to protein aggregation propensity, pivotal in the formation of amyloid fibrils and plaques that result in the neurodegeneration characteristic of prion diseases [38].

## 3. Results

### 3.1. Identification of Polymorphic Sites of the PRNP Gene in 92 Serra da Estrela Sheep

The allele sequences derived from 92 sheep were deposited in GenBank under accession numbers PV854868 to PV855051.

A total of 27 SNPs, including 20 non-synonymous SNPs, were identified (Table 1). Due to low genotype and allele frequencies, the Hardy–Weinberg exact test was used to determine if the observed genotype frequencies in the population deviate significantly from the frequencies expected under Hardy–Weinberg equilibrium (HWE), and eight SNPs, including four non-synonymous (Q171H, N176K, Q226H, and G256R), did not conform to HWE.

The haplotype distribution of the 20 nonsynonymous PRNP variants was investigated (Table 2). Variants occurring as singletons were excluded, and analysis of the other nine sites identified 13 major haplotypes. The most frequent was QALHRQNG (40.2%), whereas QALHRRNG (27.7%) and QALHRQNR (3.8%) appeared at lower proportions.

### 3.2. Estimation of Potential Scrapie Vulnerability

The genotypes of classical scrapie susceptibility are divided into five main categories, according to the three main codons (136, 154, and 171), where one corresponds to sheep highly resistant and five to sheep highly susceptible [39]. In this study 17 sheep (18.5%) had the highly resistant genotype ARR/ARR, 37 sheep (40.2%) had genotypes in the second risk class (ARR/ARH and ARR/ARQ), and 33 sheep (35.9%) in the third risk class, which are considered to have a low genetic resistance to classical scrapie and, therefore, their use in breeding programs should be avoided (Table 3). There were no sheep with genotypes in the fourth and fifth risk classes; however, five sheep showed genotypes not classified in this index (all heterozygous ARQ).

### 3.3. Impact of Nonsynonymous SNPs of the PRNP Gene in Serra da Estrela Sheep

Four different results predictions were observed, as shown in Table 4: 12 benign (M112T, H114R, A136V, L141F, H143R, Q171R, Q171K, N176K, Q189L, V213L, R223K, and Q226H), one possibly damaging (Q171H), six probably damaging (Q74S, Q101R, A121S, R154H, Y172F, and S239P), and two unknown (L245P and G256R).

**Figure 1:**
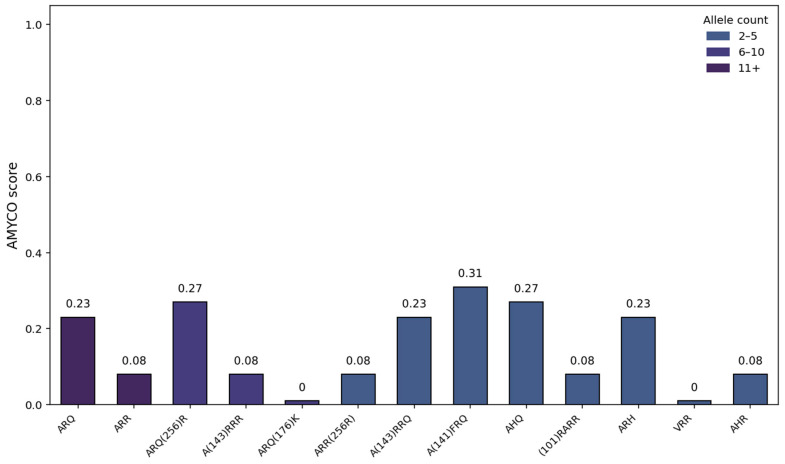
AMYCO scores (as ordered in Table 2) of the haplotypes observed in Serra da Estrela sheep.

AMYCO results are shown in Figure 1.

## 4. Discussion

Classical scrapie is a fatal disease, and the main eradication strategies include genotyping and the subsequent selection of animals carrying the ARR/ARR genotype [2,40]. To date, very limited data are available on the presence and characterisation of the *PRNP* gene in sheep of Portugal, specifically the Serra da Estrela breed. To shed light on this matter, the present study analysed the nucleotide sequence of 92 Serra da Estrela sheep and revealed 27 SNPs, 20 of which are nonsynonymous. Results show that the Serra da Estrela sheep breed presents a high frequency of the resistant ARR allele, observed in 58.7% of the sampled population, with 18.5% of individuals being homozygous (ARR/ARR). Previous studies in Portugal had similar values in the same breed as in this study; in 2010, 17.4% ARR/ARR and 42.0% ARR/ARQ were found [30] and, in 2006, 17.2% ARR/ARR and 43.8% ARR/ARQ [29]. Since frequencies of these codons have apparently remained stable throughout these last two decades, it is tempting to speculate that no animal improvement strategies regarding scrapie prevention have been implemented in the Serra da Estrela sheep breed.

Other than the 136, 154, and 171 codons, associated with classical scrapie susceptibility, this study also assessed other codons associated with resistance. Two potential resistant variants were observed, namely M112T (0.5% allele frequency) and N176K (6% allele frequency) [26]. Curiously, previous studies in Portugal or Spain did not take into consideration these two codons. However, in Italy, these two SNPs have already been reported (M112T with a frequency of up to 31.8% depending on the breed, and N176K of 4.6% in Sarda breed) [20] and, in Morocco, 2.27% frequency was observed for N176K [27]. Since current knowledge suggests these two mutations have a protective effect [26], it would be valuable to consider them in future breeding strategies and to use them as surveillance markers in upcoming studies. Although their current frequencies are very low, they can be monitored longitudinally to safeguard genetic diversity and guide future decisions. However, at present, the available data do not support diverting selection pressure away from the ARR allele toward these rare polymorphisms, especially in a breed that already shows a high frequency of ARR.

Additionally, codon 141, associated with atypical scrapie, was also evaluated. The ancestral haplotype codes for leucine (L) but an SNP in nucleotide 421 (C > T) will result in coding phenylalanine (F). This mutation has been associated with susceptibility to atypical scrapie, with AF_141_RQ and AL_141_HQ being the variants with the highest susceptibility [5,33]. In the present study we found an allele frequency of 4.3% for L141F, 2.7% for AF_141_RQ haplotype, and 2.2% for AL_141_HQ haplotype. Since there are limited data on this polymorphism in Portugal and research focuses on the main 136, 154, and 171 codons, it would be a good recommendation to have this codon included in future studies to identify susceptibility to atypical scrapie parallel to classical scrapie. This should also have an impact when applying animal improvement strategies to avoid accidentally increasing the frequency of haplotypes with increased susceptibility to atypical scrapie; as previous studies have found cases of atypical scrapie in sheep with ARR/ARR genotypes, it is worth discussion on whether increasing this genotype frequency to protect from classical scrapie will increase the cases of atypical scrapie in a country with a higher number of reports when compared with other countries.

Other nonsynonymous SNPs found in this study have been described before: Q101R (1.6% allele frequency), H143R (6.5%), and Q189L (0.5%) [41,42]. Noteworthy, to the best of our knowledge, the remaining nine nonsynonymous SNPs (G74S, H114R, A121S, Y172F, R223K, Q226H, S239P, L245P, and G256R) observed in this study have not been described before; lastly, it is of note that all these, with the exception of the SNPs at codons 245 (1.1% allele frequency), 226 (1.1%), and 256 (8.2%), were found in only one allele (0.5%).

Some of the results were marked as unknown by PolyPhen-2, which typically indicates that the algorithm was unable to predict the impact of the substitution due to insufficient sequence conservation data. This often occurs when the affected region lacks reliable multiple sequence alignment, commonly seen in proteins with low-complexity regions, repetitive sequences, or non-globular domains [37]. A likely explanation in our case is the sequence divergence between the sheep PRNP protein and its human counterpart, which serves as the reference for the tool’s predictions. AMYCO results support the current knowledge of ARR resulting in the coding of prion proteins less likely to form aggregates and, therefore, more resistant to scrapie. On the other hand, some nonsynonymous SNPs (G256R for example) might increase aggregation propensity and others (N176K for example) might decrease it. A previous study made simulations of breeding strategies that assumed a selection nucleus would be formed, where all breeding males and females, as well as replacement lambs, would be genotyped, with a stable flock of 3000 males and 120 rams; ARR/ARR rams would be preferentially used in ewes carrying the ARR allele. For a breed with similar genotype frequencies (Merino Branco), it was estimated that eight years would be needed to obtain a homozygous classical scrapie-resistant line [29]. However, the benefits of implementing a classical scrapie resistance breeding program in Portugal, where no clinical cases have been reported, must be balanced against potential drawbacks. Chief among these is the risk of increased inbreeding and reduced genetic diversity, particularly in native sheep breeds such as Serra da Estrela. Additionally, reduced selection pressure for production and adaptation traits and possible genetic correlations with scrapie resistance highlight the need for a gradual, balanced approach. Breeding programs should aim to integrate disease resistance with the preservation of genetic variability, productivity, and adaptability, rather than rushing toward full ARR homozygosity. There is also the issue of the farmers’ compliance: farmers who might see these programs as overzealous, expensive, and time-consuming. An example of a more gradual and balanced approach, given that reproductive practices in this region, predominantly would rely on a limited number of breeding males within each flock [35], involving genotyping males prior to their use in reproduction, selecting exclusively those homozygous for the AL_141_RR allele. According to this study’s results Serra da Estrela already has a 57.8% ARR allele frequency (18.5% ARR/ARR haplotype frequency), and the undesirable VRQ was not detected; this could mean that genotyping the males would ensure the increase in the AL_141_RR allele in the flocks without the need to genotype females and lambs, resulting in a slower road towards classical and atypical scrapie resistance but with better farmer compliance, safeguarding genetic variability, and ensuring preservation of the breed.

This study has some limitations that should be considered. First, both PolyPhen-2 and AMYCO were originally developed for predicting the functional effects of variants in the human *PRNP* gene, and their application to ovine sequences must be interpreted with caution. Although predictions such as the damaging effect of R154H or the reduced aggregation propensity of ARR highlight potential structural consequences, they do not fully reflect the biological complexity of scrapie. A well-known example of this complexity is the ARR genotype, which provides protection against classical scrapie but has been associated with increased susceptibility to the atypical form [43]. In our work, these tools were therefore used in an exploratory way to generate hypotheses rather than to produce definitive conclusions about susceptibility. In addition, eight SNPs identified in this study did not conform to Hardy–Weinberg equilibrium. While this may be partly explained by low allele frequencies and the limited sample size, deviations could also reflect underlying biological processes such as natural selection, genetic drift, or non-random mating. For example, Q171H, predicted by PolyPhen-2 as possibly damaging, may represent a deleterious variant under negative selection, consistent with the observed departure from equilibrium. Together, these findings suggest that some PRNP polymorphisms could be subject to selective pressures, potentially influencing prion protein function and disease susceptibility. Furthermore, the limited sample size may result in insufficient statistical power for certain low-frequency SNPs and rare alleles may have been underrepresented or missed entirely. A larger dataset covering additional flocks and regions would be recommended to validate these results, improve statistical power, and provide a more comprehensive picture of genetic diversity and scrapie susceptibility in this native Portuguese breed.

## 5. Conclusions

This study provides the most comprehensive characterisation to date of PRNP polymorphisms in Serra da Estrela sheep, identifying both known and novel variants with potential implications for scrapie susceptibility. The high frequency of the protective ARR allele, alongside the absence of the highly undesirable VRQ variant, highlights a favourable genetic profile for resistance to classical scrapie. Additionally, the identification of polymorphisms not previously reported, including those with potential protective or deleterious effects, broadens current knowledge of PRNP diversity and highlights the importance of monitoring beyond the traditionally targeted codons.

From a practical standpoint, these findings support the implementation of gradual, balanced breeding strategies that prioritise the use of ARR/ARR rams. Such an approach would enhance scrapie resistance while preserving genetic diversity and ensuring farmer compliance. By integrating genetic surveillance into breeding programs, it is possible to strengthen the long-term sustainability, health, and economic value of this culturally important Portuguese breed.

## Figures and Tables

**Table 1:** Genotype and allele frequencies of 27 PRNP polymorphisms in Serra da Estrela sheep. Each SNP shows nucleotide position and codon position, i.e., G220A is the SNP (G > A) in nucleotide 220 corresponding to the G > S amino acid mutation coded from codon 74.

*SNP*	*Genotype Frequency* *n* *(%)*			*Allele Frequency*		HWE *
***G220A***	**GG**	**GA**	AA	G	A	Yes
*G74S*	91 (98.9%)	1 (1.1%)	0 (0.0%)	183 (99.5%)	1 (0.5%)	
*A302G*	AA	AG	GG	A	G	Yes
*Q101R*	89 (96.7%)	3 (3.3%)	0 (0.0%)	181 (98.4%)	3 (1.6%)	
*T335C*	TT	TC	CC	T	C	Yes
*M112T*	91 (98.9%)	1 (1.1%)	0 (0.0%)	183 (99.5%)	1 (0.5%)	
*A341G*	AA	AG	GG	A	G	Yes
*H114R*	91 (98.9%)	1 (1.1%)	0 (0.0%)	183 (99.5%)	1 (0.5%)	
*G361T*	GG	GT	TT	G	T	Yes
*A121S*	91 (98.9%)	1 (1.1%)	0 (0.0%)	0 (0.0%)	1 (0.5%)	
*C407T*	CC	CT	TT	C	T	Yes
*A136V*	90 (97.8%)	2 (2.2%)	0 (0.0%)	182 (98.9%)	2 (1.1%)	
*C421T*	CC	CT	TT	C	T	Yes
*L141F*	84 (91.3%)	8 (8.7%)	0 (0.0%)	176 (95.7%)	8 (4.3%)	
*A428G*	AA	AG	GG	A	G	Yes
*H143R*	81 (88.0%)	10 (10.9%)	1 (1.1%)	172 (93.5%)	12 (6.5%)	
*G461A*	GG	GA	AA	G	A	Yes
*R154H*	86 (93.5%)	5 (5.4%)	1 (1.1%)	177 (96.2%)	7 (3.8%)	
*C511A*	CC	CA	AA	C	A	Yes
*Q171K*	91 (98.9%)	1 (1.1%)	0 (0.0%)	183 (99.5%)	1 (0.5%)	
*A512G*	AA	AG	GG	A	G	Yes
*Q171R*	34 (37.0%)	41 (44.6%)	17 (18.5%)	109 (59.2%)	75 (40.8%)	
*G513T*	GG	GT	TT	G	T	No
*Q171H*	88 (95.7%)	2 (2.2%)	2 (2.2%)	178 (96.7%)	6 (3.3%)	
*A515T*	AA	AT	TT	A	T	Yes
*Y172F*	91 (98.9%)	1 (1.1%)	0 (0.0%)	183 (99.5%)	1 (0.5%)	
*G525A*	GG	GA	AA	G	A	Yes
*175 Q*	91 (98.9%)	1 (1.1%)	0 (0.0%)	183 (99.5%)	1 (0.5%)	
*C528A*	CC	CA	AA	C	A	No
*N176K*	84 (91.3%)	5 (5.4%)	3 (3.3%)	173 (94.0%)	11 (6.0%)	
*A566T*	AA	AT	TT	A	T	Yes
*Q189L*	91 (98.9%)	1 (1.1%)	0 (0.0%)	183 (99.5%)	1 (0.5%)	
*G594C*	GG	GC	CC	G	C	No
*198G*	91 (98.9%)	0 (0.0%)	1 (1.1%)	182 (98.9%)	2 (1.1%)	
*G630A*	GG	GA	AA	G	A	Yes
*210E*	91 (98.9%)	1 (1.1%)	0 (0.0%)	183 (99.5%)	1 (0.5%)	
*G637C*	GG	GC	CC	G	C	No
*212V*	91 (98.9%)	0 (0.0%)	1 (1.1%)	182 (98.9%)	2 (1.1%)	
*G668A*	GG	GA	AA	G	A	Yes
*R223K*	91 (98.9%)	1 (1.1%)	0 (0.0%)	183 (99.5%)	1 (0.5%)	
*G678C*	GG	GC	CC	G	C	No
*Q226H*	91 (98.9%)	0 (0.0%)	1 (1.1%)	182 (98.9%)	2 (1.1%)	
*A691C*	AA	AC	CC	A	C	Yes
*231R*	58 (63.0%)	27 (29.3%)	7 (7.6%)	143 (77.7%)	41 (22.3%)	
*C711G*	CC	CG	GG	C	G	No
*237L*	56 (60.9%)	13 (14.1%)	23 (25.0%)	125 (67.9%)	59 (32.1%)	
*T715C*	TT	TC	CC	T	C	Yes
*S239P*	91 (98.9%)	1 (1.1%)	0 (0.0%)	183 (99.5%)	1 (0.5%)	
*T723C*	TT	TC	CC	T	C	No
*241P*	91 (98.9%)	0 (0.0%)	1 (1.1%)	182 (98.9%)	2 (1.1%)	
*T734T*	TT	TC	CC	T	C	Yes
*L245P*	90 (97.8%)	2 (2.2%)	0 (0.0%)	182 (98.9%)	2 (1.1%)	
*G766A*	GG	GA	AA	G	A	No
*G256R*	83 (90.2%)	3 (3.3%)	6 (6.5%)	169 (91.8%)	15 (8.2%)	

* HWE: Hardy–Weinberg equilibrium.

**Table 2:** Haplotype frequencies of nonsynonymous SNPs of *PRNP* gene in Serra da Estrela sheep. SNPs with allele frequency of one (1) are aggregated in the “Others” category.

Haplotypes	A302G	C407T	C421T	A428G	G461A	A512G	G513T	C528A	G766A	
	Q101R	A136V	L141F	H143R	R154H	Q171R	Q171H	N176K	G256R	*N* = 184
Hap 1	Q	A	L	H	R	Q		N	G	74 (0.4022)
Hap 2	Q	A	L	H	R	R		N	G	51 (0.2772)
Hap 3	Q	A	L	H	R	Q		N	R	7 (0.0380)
Hap 4	Q	A	L	R	R	R		N	G	6 (0.0326)
Hap 5	Q	A	L	H	R	Q		K	G	8 (0.0435)
Hap 6	Q	A	L	H	R	R		N	R	5 (0.0272)
Hap 7	Q	A	L	R	R	Q		N	G	5 (0.0272)
Hap 8	Q	A	F	H	R	Q		N	G	3 (0.0163)
Hap 9	Q	A	L	H	H	Q		N	G	2 (0.0109)
Hap 10	R	A	L	H	R	R		N	G	2 (0.0109)
Hap 11	Q	A	L	H	R		H	N	G	2 (0.0109)
Hap 12	Q	V	L	H	R	R		N	G	2 (0.0109)
Hap 13	Q	A	L	H	H	R		N	G	2 (0.0109)
Others										15

**Table 3:** Genotypes for the 136, 154, and 171 codons in Serra da Estrela sheep, sorted by classical scrapie risk class. Adapted [39].

Risk Class	Genotype	*n* (%)
1	ARR/ARR	17 (18.48)
2	ARR/ARH	2 (2.17)
	ARR/ARQ	35 (38.04
3	ARQ/ARH	1 (1.09)
	ARQ/AHQ	1 (1.09)
	AHQ/AHQ	1 (1.09)
	ARH/ARH	2 (2.17)
	ARQ/ARQ	28 (30.43)
other	ARQ/VRR	2 (2.17)
	ARQ/ARK	1 (1.09)
	ARQ/AHR	2 (2.17)

**Table 4:** PolyPhen-2 prediction of effect of amino acid substitutions of PRNP nonsynonymous SNPs (AA_1_: ancestral amino acid; AA_2_: amino acid derived from nonsynonymous SNP).

Position	AA_1_	AA_2_	Score	Prediction
74	G	S	1.000	Probably Damaging
101	Q	R	0.621	Probably Damaging
112	M	T	0.007	Benign
114	H	R	0.331	Benign
121	A	S	1.000	Probably Damaging
136	A	V	0.156	Benign
141	L	F	0.411	Benign
143	H	R	0.241	Benign
154	R	H	0.997	Probably Damaging
171	Q	R	0.005	Benign
171	Q	K	0.040	Benign
171	Q	H	0.586	Possibly Damaging
172	Y	F	0.999	Probably Damaging
176	N	K	0.002	Benign
189	Q	L	0.277	Benign
213	V	L	0.002	Benign
223	R	K	0.000	Benign
226	Q	H	0.199	Benign
239	S	P	0.988	Probably Damaging
245	L	P	not available	Unknown
256	G	R	not available	Unknown

## Data Availability

The original contributions presented in this study are included in the article/Supplementary Materials. Further inquiries can be directed to the corresponding author.

## Supplementary Materials

The following supporting information can be downloaded at: https://www.mdpi.com/article/10.3390/ani15182750/s1, Figure S1: AMYCO scores for haplotypes observed only once.
