# Supplementary figures and images for: Mapping PRNP Polymorphisms in Portuguese Serra da Estrela Ovine Populations: Insights into Scrapie Susceptibility and Farm Animal Improvement
Source: Animals (Basel). 2025 Sep 20;15(18):2750. doi: 10.3390/ani15182750 (PMC12466367; doi:10.3390/ani15182750)

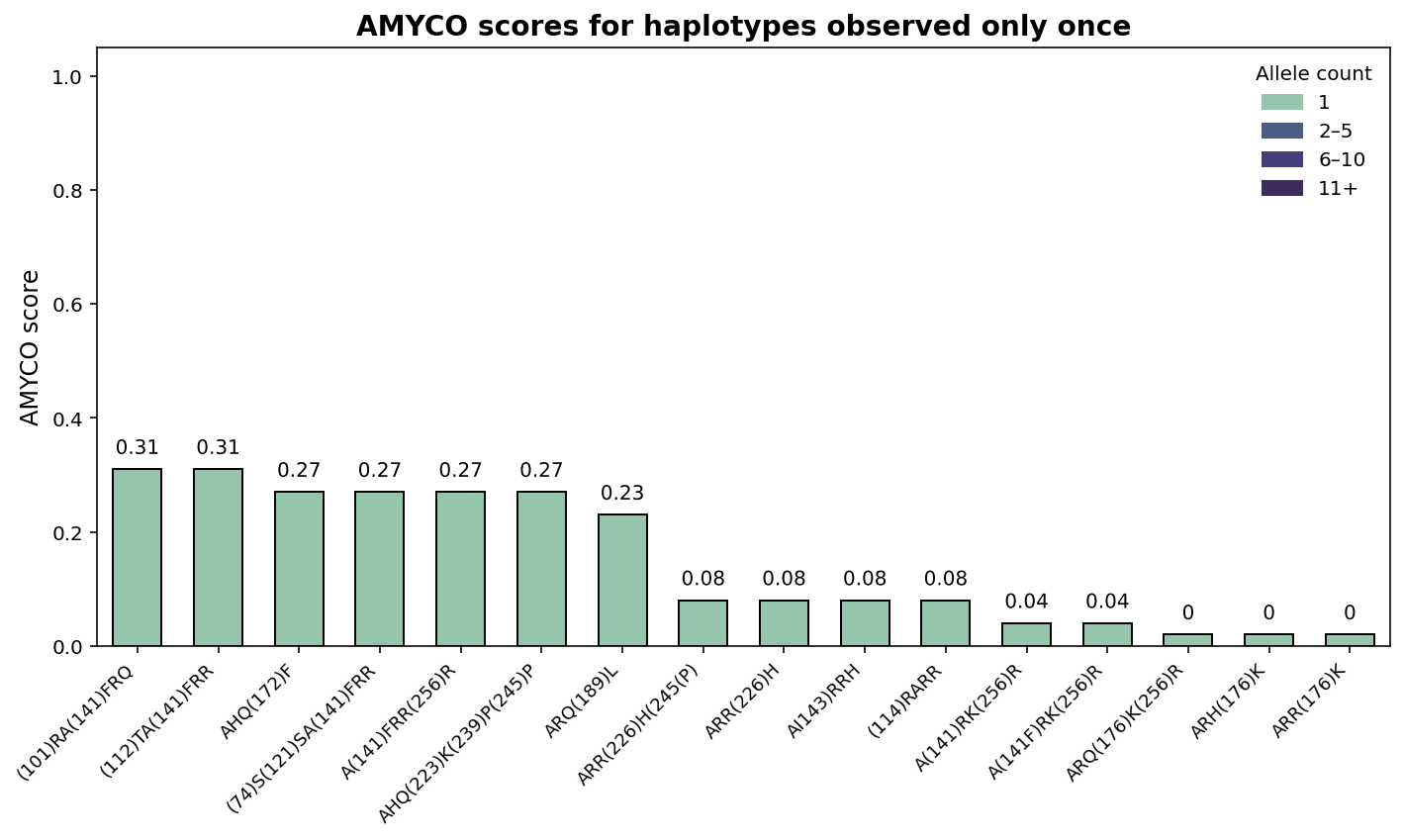

Supplement: Supplementary file 1 [file animals-15-02750-s001.zip › Supplementary Figure S1_others_AMYCO.png]
